# Sulphate of Bebeeria in Facial Neuralgia

**Published:** 1866-06

**Authors:** 


Sulphate of Bebeeria in Facial Neuralgia.—The following prescription is highly recommended in this distressing affection : Sulphate of bebeeria, one drachm ; dilute sulphuric acid, one drahm ; tincture of oranges, two drachms ; distilled water, one ounce and a half: one drachm to be taken in water every two hours.

